# Variation in origin of the long head of the biceps brachii tendon in a cadaver: a case report: Erratum

**DOI:** 10.1097/MD.0000000000011198

**Published:** 2018-06-15

In the article, “Variation in origin of the long head of the biceps brachii tendon in a cadaver: A case report”^[1]^, which appeared in Volume 97, Issue 20 of *Medicine*, the citation in the third sentence appeared incorrectly and the sentence should have appeared as “Thus, the LHB tendonsuperior glenoid labrum complex has a stabilizing mechanism for the humeral head in the shoulder joint.^[2–6]^”
